# Interferon-gamma release assays for the tuberculosis serial testing of health care workers: a systematic review

**DOI:** 10.1186/1745-6673-7-6

**Published:** 2012-06-18

**Authors:** Felix C Ringshausen, Anja Schablon, Albert Nienhaus

**Affiliations:** 1 Department of Respiratory Medicine, Hannover Medical School, Hannover, Germany; 2 Institute for Health Services Research in Dermatology and Nursing, Hamburg-Eppendorf University Hospital, Hamburg, Germany; 3 Department of Occupational Health Research, Institution for Statutory Accident Insurance and Prevention in Health and Welfare Services, Hamburg, Germany

**Keywords:** Interferon-γ release assay, Health care workers, Latent tuberculosis infection, Occupational disease, Serial testing, Tuberculosis, Within-subject variability

## Abstract

**Background:**

Interferon-gamma release assays (IGRAs) are increasingly used in the tuberculosis (TB) screening of health care workers (HCWs). However, comparatively high rates of conversions and reversion as well as growing evidence of substantial within-subject variability of interferon-gamma responses complicate their interpretation in the serial testing of HCWs.

**Methods:**

We conducted a systematic review on the repeat use of the two commercial IGRAs, the QuantiFERON-TB Gold or In-Tube version (QFT) and the T-SPOT.TB (T-SPOT), in the serial testing and its with-subject variability among HCWs in order to provide guidance on how to interpret serial testing results in the context of the periodic screening of subjects with an increased occupational risk of latent TB infection (LTBI) in countries with low and intermediate TB incidence rates. The Medline, Embase, and Cochrane databases were searched without restrictions. Retrieved articles were complemented by additional hand searched records. Only studies that used commercial IGRAs among HCWs apart from contact and outbreak investigations and those fulfilling further predefined criteria were included.

**Results:**

Overall, 20 studies, five using the T-SPOT and 19 using the QFT assay, were included. Fifteen studies met eligibility criteria for serial testing and five studies for within-subject variability. Irrespective of TB incidence rates in the study’s country of origin, reversion rates were consistently higher than conversion rates (range 22–71% vs. 1–14%). Subjects with baseline results around the diagnostic threshold were more likely to show inconsistent results on retesting. The within-subject variability of interferon-gamma responses was considerable across all studies systematically assessing it.

**Conclusions:**

On the basis of reviewed studies we advocate using a borderline zone from 0.2–0.7 IU/ml for the interpretation of repeat QFT results in the routine screening of HCWs with an increased LTBI risk. Subjects with QFT results within this borderline zone, with suspected fresh infection, and those who are considered for preventive chemotherapy should be retested with the QFT within a period of about four weeks before preventive chemotherapy is recommended. However, the available data regarding the use of the T-SPOT in the serial testing of HCWs is remarkably limited and warrants further research.

## Background

Health care workers (HCWs) may have an increased risk of occupational tuberculosis (TB) infection due to possible nosocomial exposure [1-4]. Therefore, exposed HCWs are subject to periodic TB screening with a view to identifying infection at an early stage and preventing the development of active TB by means of preventive chemotherapy [5,6]. In the absence of clinical evidence of active TB, latent TB infection (LTBI) is understood by current consensus to be proven by a *Mycobacterium tuberculosis*-specific T-cell-mediated adaptive immune response, either by a positive tuberculin skin test (TST) or a positive interferon-γ release assay (IGRA) [7]. However, that does not necessarily have to mean that live and augmentable mycobacteria exist. In Germany, section 4 of the regulation on occupational safety and health (OSH) prescribes regular compulsory screening of employees in infectious diseases, respiratory medicine, and in laboratories insofar as they come into regular contact with people suffering or suspected of suffering from TB, or possibly coming into contact with infectious or contaminated material [8]. Usually, these OSH screenings are performed at one-year to three-year intervals. In addition to identifying LTBI as an irregular physical condition by the terms of OSH legislation, these screenings are aimed at establishing the rate of new TB infections as a measure of the effectiveness of the preventive measures implemented and at reducing the risk of a HCW to develop active TB, e.g. by prescribing preventive chemotherapy.

Despite its known limitations, in particular its cross-reactivity after vaccination with the attenuated *Mycobacterium bovis* live strain of bacillus Calmette-Guérin (BCG) and infection with nontuberculous mycobacteria, the TST, which is more than 100 years old, has been the only way to diagnose LTBI for decades [9,10]. Its use in serial testing is complicated by sensitization as a result of repeated use (the so-called “boosting” phenomenon) [11]. In this respect the two IGRAs, the QuantiFERON-TB Gold and In-Tube version (QFT) and T-SPOT.TB (T-SPOT), which have been commercially available for a number of years, offer distinct advantages. They have now established as alternatives to the TST [12]. IGRAs are ex-vivo blood tests that avoid the sensitization of the immune system to mycobacterial antigens in serial testing (“boosting”). Even among BCG-vaccinated HCWs, IGRAs have a high specificity and correlate well to the occupational risk of TB exposure [13-15]. They also possess certain logistical advantages over the TST in that a second appointment to read the result is no longer required.

However, IGRAs are also subject to a certain biological and technical variability. Thus, they must be considered as dynamic tests [14,16,17]. Different borderline zones (“gray zones”) have been proposed for defining “genuine” conversions and reversions in order to improve the interpretation of test results as part of IGRA serial testing [12,17-21]. As there is no gold standard for diagnosing LTBI, the definition of an appropriate borderline zone is challenging and an issue of ongoing scientific debate. Data on the prediction of active TB after conversion, reversion, or persistently positive IGRA results may help to determine these borderline zones, but this data is not readily available [22].

We performed a systematic review on the repeat use of commercial IGRAs among HCWs, either in serial testing studies or in studies on the within-subject variability of interferon-(IFN)-γ responses (as determined by commercial IGRAs), in order to inform policies and practices related to the TB serial testing of HCWs in countries with low and intermediate TB incidence rates.

## Methods

### Search strategy, study selection, and eligibility

This systematic review was conducted according to the guidelines of the Preferred Reporting Items for Systematic Reviews and Meta-Analyses (PRISMA) statement [23]. We searched the Medline, Embase, and Cochrane databases without restrictions regarding language or study design though 15 Mar 2012. The following search string was used: ("Tuberculosis" OR "TB infection") AND ("interferon-gamma" OR “gamma-interferon” OR "interferon" OR “interferon-gamma release assay” OR “IGRA” OR "Quantiferon" OR "T-SPOT") AND ("health care workers" OR "healthcare workers" OR "health-care workers" OR "health personnel"). In addition reference lists, citations of previous reviews, and abstracts from conference proceedings were hand searched. In case of doubt authors of original studies were contacted to obtain additional information. Only original studies (research articles or letters containing original data) among HCWs or those containing a significant proportion of HCWs (> 50% of the study population) that were conducted apart from (cross-sectional) LTBI prevalence, contact tracing, or outbreak investigations and repeatedly used one of the two or both commercially available IGRAs on the same group of subjects (at least twice with the same assay; longitudinal or serial testing design) were considered eligible. Case reports, case series, comments, editorials, reviews, cost effectiveness analyses, studies not following the manufacturers’ instructions (e.g. studies using prolonged incubation or freezing of cells), and studies involving less than 10 subjects or any kind of therapeutic intervention such as treatment for LTBI or active TB (i.e. studies using an IGRA for the monitoring of treatment response) were excluded.

For an optimal assessment of the behavior of IGRAs in serial testing, these studies had to be performed among HCWs with a low or intermediate occupational TB risk, i.e. not in a context of point source exposure and not within a TB contact or outbreak investigation, and with a follow-up for repeat IGRA testing of at least four weeks. In principle, the same preconditions applied to studies on the within-subject variability of IFN-γ responses as determined by commercial IGRAs. The risk of exposure to TB in the course of the study is particularly important for the results of any such study. Those studies performed in countries with an intermediate or high incidence of TB should therefore have been conducted over as short a period as possible (less than four weeks) in order to avoid conversions due to fresh TB infection. Furthermore, consistent, well defined, and controlled or controlled-modified test conditions should have been applied and additional quality assurance measures should have been implemented. Studies with subjects who have had a TST within the past three months were explicitly excluded due to evidence that a recent TST may influence IGRA results by the “boosting” of IFN-γ responses. Apparently, this applies if sensitization to mycobacterial antigens preexists [20,24,25].

### Data extraction, data analysis, and quality assessment

Two reviewers (AS and FCR) independently assessed articles for eligibility. Disagreement was resolved by consensus. Data extraction was performed using a data extraction form by one reviewer (FCR) and confirmed by a second reviewer (AS). In serial testing studies the outcomes of interest were: recruitment period, country and epidemiological setting of the study according to the World Health Organization (WHO) [26], IGRA(s) used, interval(s) between repeat IGRA testing, number of included subjects, rate of positive results at baseline, rate of reversions, and rate of conversions. In studies on the IGRA within-subject variability the outcomes of interest were: country and epidemiological setting of the study according to the WHO, IGRA(s) used, number of included subjects and total number of tests conducted, time points at which IGRAs were performed, and results with respect to the observed IGRA within-subject variability.

Weighted mean incidence rates were calculated for reversions and conversions. The completeness of reported outcome parameters, the reporting of quantitative follow-up IGRA results stratified according to the quantitative baseline results, and a prospective study design were considered as quality indicators. Due to the apparent heterogeneity of study designs and study quality, time intervals between repeat IGRAs, included study populations, epidemiological settings, and the applied assays, performing pooled analysis (meta-analysis) was considered inappropriate.

## Results

**Figure 1:**
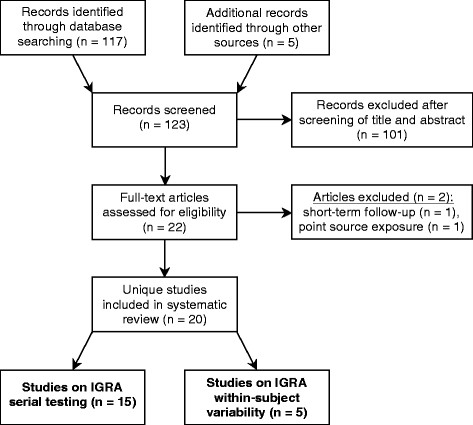
**Flow diagram for study selection.** Abbreviation: IGRA = interferon-γ release assay.

Figure 1 summarizes the process of study selection. Finally, after excluding two full-text articles that had been assessed for eligibility in detail [27,28], 20 studies were included in the review. Of those, 15 studies met eligibility criteria for the serial testing analysis [17,29-42], while 5 studies met eligibility criteria for the within-subject variability analysis [20,21,24,43,44].

### Studies on IGRA serial testing

Table 1 provides an overview of publications to date on the serial testing of HCWs using commercial IGRAs by the incidence of TB in the study’s country of origin and summarizes their most important characteristics and results. Overall, these studies included 6605 subjects. Reversion rates ranged from 22.1 to 71.4%, while conversion rates ranged from 0.7 to 14.4%. The weighted mean incidence rate of QFT reversions based on 3849 subjects from 9 studies from countries with low and intermediate TB incidence rates [30,33-35,37-40,42] was 33.6% (95% confidence interval 29.6 – 37.7). The weighted mean incidence rate of QFT conversions based on 6072 subjects from 12 studies from countries with low and intermediate TB incidence rates [29,30,32-34,36-42] was 5.1% (95% confidence interval 4.5 – 5.7). The number of subjects included in the two studies using the T-SPOT assay was comparatively low (overall 1463 subjects) [31,34]. Only one study reported the reversion rate for the T-SPOT [34]. Based on their retrospective study design [35,41] and the incompleteness of data reporting [29,31-34,36] eight studies were considered to be of minor quality, while seven studies were considered to be of superior quality [17,30,37-40,42].

**Table 1:** Characteristics and results of studies on serial testing using IGRAs

Study (Year of publication)	Recruitment period	Country	IGRA(s) used	Time between IGRAs	N*	Initially positive IGRAs^#^(%)	IGRA reversions (%)	IGRA conversions (%)
Studies in countries with a high incidence of TB (≥ 100 per 100,000 population and year)								
Pai et al. (2006) [17]	2004	India	QFT	18 months	216	38/216 (17.6)	9/38 (23.7)	18/178 (10.1)
Studies in countries with an intermediate incidence of TB (≥ 20 and < 100 per 100,000 population and year)								
Chee et al. (2009) [31]	2005–2007	Singapore	T-SPOT	1 year	182	-^†^	-^†^	9/182 (4.9)
Lee et al. (2009) [32]	2007	South Korea	QFT^‡^	1 year	169	23/169 (13.6)	-**	21/146 (14.4)
Yoshiyama et al. (2009) [30]	2003, 2005	Japan	QFT^‡^	2–4 years	311	27/311 (8.7)^††^	13/31 (41.9)^††^	6/287 (2.1)^††^
Park et al. (2010) [36]	2008–2009	South Korea	QFT	1 year	275	29/275 (10.5)	-**	14/244 (5.7)
Torres Costa et al. (2011) [40]	2007–2009	Portugal	QFT	1 year	670	208/670 (31.0)	46/208 (22.1)	51/462 (11.0)
Rafiza et al. (2012) [42]	2009–2010	Malaysia	QFT	1 year	769	64/769 (8.3)	19/64 (29.7)	69/704 (9.8)
Studies in countries with a low incidence of TB (< 20 per 100,000 population and year)								
Pollock et al. (2008) [29]	2006	USA	QFT^‡^	1–7 months	43	-^†^	-^†^	2/43 (4.7)
Zwerling et al. (2009)^¶^[33]	2007–2008	Canada	QFT	1 year	60	4/60 (6.7)	2/4 (50.0)	4/56 (7.1)
Belknap et al. (2010)^¶^[34]	Unknown	USA	QFT	6 months	1281	50/1281 (3.9)	20/50 (40.0)	44/1169 (3.8 )
			T-SPOT		68/1281 (5.3)	68/1281 (5.3)	36/68 (52.9)	44/1117 (3.9)
Gandra et al. (2010) [35]^##^	2008	USA	QFT	4 weeks	135	-^##^	66/135 (48.9)	-^##^
Ringshausen et al. (2010) [37]	2005–2008	Germany	QFT	18 weeks	182	18/182 (9.9)	6/18 (33.3)	3/162 (1.9)
Schablon et al. (2010) [38]	2006–2009	Germany	QFT	1 year	287	42/287 (14.6)	13/42 (31.0)	15/245 (6.1)
Schablon et al. (2011) [39]	2008–2009	Germany	QFT	1 year	154	2/154 (1.3)	1/2 (50.0)	1/152 (0.7)
Fong et al. (2012) [41]^§^	2007–2010	USA	QFT	1 year	1871	-^§^	10/14 (71.4)^§^	52/1857 (2.8)

* Number of subjects with IGRA test repeats in the course of the study.

^#^ Initially positive IGRA results in the cohort with IGRA test repeats.

^†^ Only subjects with initially negative IGRA results were retested.

^‡^ QuantiFERON-TB Gold.

** Reversion rate not reported.

^††^ Over the course of the study 139 subjects were retested thrice. Conversion and consecutive reversion occurred in three subjects, while reversion and consecutive conversion occurred in one subject.

^¶^ Published abstract.

^##^ Retrospective chart review. Only subjects with initially positive IGRA results were retested.

^§^ Retrospective chart review. According to the institution’s occupational health policy, health care workers with positive IGRA results were not subject to annual repeat testing. Retesting of initially IGRA-positives was performed on an individual basis only. Hence, the remainders of the 486 initially QFT-positive subjects (486/7374; 6.6%) were either advised LTBI therapy, declined retesting, or were lost to follow-up. Of the subjects with IGRA conversions on retesting, ten subjects were retested for a third time, of whom eight (80%) showed a reversion back to a negative IGRA result.

Abbreviations: IGRA = interferon-γ release assay; QFT = QuantiFERON-TB Gold In-Tube; TB = Tuberculosis; T-SPOT = T-SPOT.TB.

Even though the included serial testing studies differed substantially in their design and epidemiological setting, what they all had in common is that inconsistent IGRA serial testing results (conversions and reversions) occurred fairly frequently and that even in countries with an intermediate or high incidence of TB, reversions of positive IGRA results were observed more frequently than conversions of negative results ( 1). Moreover, in studies reporting (stratified) quantitative QFT results, the likelihood of conversions and reversions of QFT results increased in subjects with initial test results close to the diagnostic threshold, generally within a borderline zone between 0.2 to 0.7 IU/ml [37-42]. However, published serial testing studies reporting quantitative T-SPOT results for analysis were unavailable within the searched databases.

### Studies on the within-subject variability of the IFN-γ response

So far, there were only few well-conceived studies that have investigated the (biological) within-subject variability or the influence of technical issues on the reproducibility of the *Mycobacterium tuberculosis*-specific IFN-γ response [20,21,24,43,44]. Table 2 summarizes the studies on the within-subject variability of IFN-γ responses that have been published to date and that met the eligibility criteria mentioned above. However, the number of subjects tested in the available studies was low (overall 219 subjects) and ranged from 14 to 117 subjects. These studies were carried out almost exclusively on HCWs. Remarkably, studies from countries with a low incidence of TB [24,39,44] as well as studies comparing the two commercially available IGRAs [20,24,44] have rarely been published. Differences in the statistical assessment of within-subject variability handicap comparability (Table 2), but what all the above-mentioned studies had in common was that the amount of observed within-subject variability was considerable and, again, that conversions or reversions of IGRA results were more likely to be found if the initial test result was close to the diagnostic cut-off [20,21,24,43,44].

**Table 2:** Characteristics and results of studies on within-subject variability of the IFN-γ response

Study (Year of publication)	Country	IGRA(s) used	Subjects (total number of tests)	Visits (Days)	Summary of results
Studies in countries with a high incidence of TB (≥ 100 per 100,000 population and year)*					
Veerapathran et al. (2008) [21]	India	QFT	14 (56)	0, 3, 9, 12	Over a two-week period, two out of 14 subjects (14%) had a QFT reversion. Overall, reproducibility of the quantitative results was moderate. A non-significant 30% reduction in mean IFN-y response was observed between visits. An increase of up to 16% in IFN-γ concentration was within the expected within-subject variability.
Van Zyl-Smit et al. (2009) [20]	South Africa	QFT T-SPOT	26^#^ (88)	0, 7, 14, 21	Over a three-week period seven out of 26 volunteers had a conversion or a reversion (1x QFT, 6x T-SPOT). A change in mean IFN-γ response of ± 80% (QFT) or ± 3 SFCs accounted for 95% of the within-subject variability.
Detjen et al. (2009) [43]	South Africa	QFT	27 (54)	0, 3	Over a three-day period no qualitative changes in the QFT results were noted in 15 subjects, but a partly substantial within-subject variability in IFN-γ response was observed (intra-class correlation = 0.80).
Studies in countries with a low incidence of TB (< 20 per 100,000 population and year)					
Belknap et al. (2009)^†^[24]	USA	QFT T-SPOT	117 (234)	0, 7–21	Over a three-week period seven out of 117 (6%) and eight out of 105 (8%) had a conversion or reversion with QFT or T-SPOT respectively.
Ringshausen et al. (2011) [44]	Germany	QFT T-SPOT	35 (158)	0, 7, 14, 21, 28	Changes of ± 70% (QFT) and ± 60% (T-SPOT) in mean IFN-γ response accounted for 95% of the within-subject variability. Inconsistent results were significantly more frequent with QFT (29%) than with T-SPOT (9%).

* In studies conducted in countries with a high TB incidence occult TB exposure during the study cannot be ruled out with certainty.

^#^ Health care workers and healthy (“low risk”) subjects.

^†^ Published abstract with updated preliminary results presented by Daley C. (on behalf of the CDC TB Epidemiological Studies Consortium). Evaluation of Interferon-γ Release Assays in the Diagnosis of Latent TB Infection in U.S. Healthcare Workers: Preliminary Results of Task Order #18. May 31 2009; 2nd Global Symposium on IGRAs, Dubrovnik, Croatia.

Abbreviations: IGRA = interferon-γ release assay; IFN-γ = interferon-γ; QFT = QuantiFERON-TB Gold In-Tube; SFC = spot-forming cell; TB = tuberculosis; T-SPOT = T.SPOT.TB.

## Discussion

Despite the remarkable amount of publications on the two commercially available IGRAs, their implementation in recommendations and guidelines [5,12,45-49], and their widespread use in daily clinical practice, only a few studies have been published to date on their use in the serial testing of HCWs [17,29-42] and on their within-subject variability [20,21,24,43,44]. Two other recently published systematic reviews identified a mere ten original studies on serial testing and four on within-subject variability that corresponded to previously defined selection criteria and quality indicators [15,25]. The medical literature available suggests that conversions and reversions occurring in IGRA serial testing are due to the considerable biological (within-subject) and technical variability of the IFN-γ response. The test manufacturers’ dichotomous cut-off values seem to be only partly appropriate for the interpretation of repeat IGRA results. A current review by Zwerling and colleagues on the TB screening of HCWs using IGRAs found existing data to be insufficient to make a general recommendation regarding their use instead of the TST in serial testing [15]. However, in our own experience, in a country with a low incidence of TB and a high BCG vaccination coverage the use of an IGRA instead of the TST may safely reduce the prevalence of positive test results and thereby the number of HCWs who require a chest X-ray to rule out active TB [50].

Torres Costa and colleagues, who in Portugal carried out repeat testing of the largest European cohort of HCWs to date, found a close relation between the IFN-γ response of initial and follow-up QFT results. QFT results in a borderline zone between 0.2 and 0.7 IU/ml had, when the test was repeated, undergone an especially frequent qualitative change (i.e. a change from a negative to a positive result and vice versa; 49.0% vs. 8.7% of subjects with a QFT result of < 0.2 or > 0.7 IU/ml) [40]. Furthermore, their study also revealed that using a borderline zone between 0.2 und 0.7 IU/ml could reduce the rate of QFT conversions and reversions significantly and safely. The conversion rate was reduced from 11.0% to 3.6% and the reversion rate from 22.1% to 5.2%. This observation is in accordance with the findings of two current studies of German HCWs by our own working group. Using this borderline zone, the conversion rates were reduced from 1.9% to 0.6% and from 6.1% to 2.6% and the reversion rates from 33.3% to 16.7% and from 32.6% to 15.4%, respectively [37,38]. No cases of active TB occurred during the follow-up period of more than two years. In one of the two studies in which 182 HCWs underwent repeat QFT tests we were able to show that along with increasing age and the extent of the initial IFN-γ response, a positive TST both in the past and at the time of the current evaluation was a significant predictor of a persistently positive IFN-γ response in serial testing [37]. These findings have largely been confirmed by a recent study by Gandra and colleagues in another low TB incidence country (USA) [35].

In a further recent study of our own we showed that the within-subject variability of the IFN-γ response of both IGRAs in middle-aged HCWs (mean 42 ± 10.5 years old) with a low to intermediate risk of TB exposure over a period of four weeks was quite considerable [44]. Changes in mean IFN-γ response of about ± 70% (QFT) and ± 60% (T-SPOT) accounted for 95% of the within-subject variability. With dichotomous interpretation of test results, inconsistent results occurred with significantly greater frequency with the QFT (29%) than with the T-SPOT (9%; p < 0.001*)*. The statistical phenomenon of regression towards the means led to a significant reduction in the mean IFN-γ response of both IGRAs by about −25% from week to week. Applying borderline zones between 0.2 to 0.7 IU/ml (QFT) and between 4 to 8 spot-forming cells (SFCs; T-SPOT) reduced primarily the rate of QFT reversions (from 50% to 17%). These results are comparable with those of a South African study in which changes of ± 80% (QFT) and ± 3 SFCs (T-SPOT) were responsible for 95% of the within-subject variability ( 2) [20]. Discordant results for the two IGRAs occurred in a total of 9% (8/88) of subjects and were thus similar in frequency to our own findings (13%, 20/158 subjects) [20,44]. The three studies conducted in countries with a high TB incidence (India, South Africa) can only to a limited extent be compared with studies conducted in TB low incidence countries (Germany, USA), because they included only a small number of subjects with IGRA results close to the diagnostic threshold and had high overall rates of positive IGRA results of between 40% and 57% and large maximum individual variation ranges of the absolute IFN-γ response (8.41 to 11.11 IU/ml for the QFT) [20,21,43].

Another study by our own working group investigated the specificity and the negative predictive value of the QFT among trainee nurses and HCWs. The average age of study participants was 23 ± 5.8 years. In this cohort with a very low risk of previous TB exposure there was, with respect to the qualitative findings, only a low within-subject variability of IFN-γ responses over a one-year period. Out of a total of 154 subjects who underwent a repeat test, two were positive in the first test. One of the two remained positive in the second test, whereas the other showed a reversion. Of the 152 trainees who were negative in the first test, 151 had consistently negative QFT results (99%). The one subject with a conversion showed an increase in IFN-γ concentration from 0.01 to 0.67 IU/ml. Applying the borderline zone from 0.2 to 0.7 IU/ml, this increase would not be counted as a conversion. This individual had no TB infection risk. Overall, the quantitative results were mainly stable at < 0.1 IU/ml and emphasized the high specificity of the QFT in this young cohort with a low risk of TB exposure. None of the trainees developed TB during the two-year observational period, indicating a negative predictive value of 100% among this population.

In summary, IGRA reversions have been observed more frequently than conversions in serial testing studies. A possible explanation for this observation could be the frequently inevitable statistical phenomenon of regression towards the means, which occurs wherever repeat measurements are performed of the same individual with a random measurement error, i.e. with an unsystematic spread around a true mean [51]. Taking this phenomenon into account is especially important if subjects are categorized or stratified on the basis of certain initial measurements, e.g. if the effect of a therapy or the incidence of a disease is estimated on the basis of repeat measurements of surrogate parameters. As the effect of regression towards the means increases in proportion to the variability of the method employed it can be reduced by increasing the number of independent initial measurements, i.e. by an approximation to the “true” mean. However, this approach will rarely be applicable in practice. Hence, the definition of a borderline zone for the interpretation of serial measurements is an appropriate alternative.

Our review is subject to limitations. The number of eligible and accessible studies as well as their quality and the number of included subjects were limited. Furthermore, we did not systematically assess risk factors for conversion, reversion, or persistent positivity of IGRA responses, which recently have been reported in detail somewhere else [15].

Finally, it should be emphasized that even a negative IGRA result can never rule out either active TB or LTBI with absolute certainty. Moreover, clinical information on the individual risk of exposure and the susceptibility to TB along with recent or previous TST or IGRA results should always be taken into account in the overall context. The safety of a borderline zone for the interpretation of IGRA serial results with regard to the progression toward active TB may not be inferred from studies on within-subject variability because these studies generally cover a limited follow-up period. Further studies on large cohorts of HCWs with sufficiently long follow-up periods of more than two years are required to define more precisely the prognosis of conversions, reversions, and persistently positive IGRA results.

## Conclusion

So far, the published studies on IGRA serial testing and on the within-subject variability of the IFN-γ response among HCWs in countries with a low or intermediate incidence of TB demonstrate convincingly that subjects with a QFT result in a borderline zone between 0.2 to 0.7 IU/ml are significantly more likely to show an inconsistent result on retesting [37-40,42,44]. Furthermore, applying this borderline zone to the interpretation of QFT results in the serial testing of German HCWs appears to be safe because none of the 623 HCWs in our own studies developed active TB in the follow-up period of more than two years [37-39]. According to the current literature, the experience regarding the use of the T-SPOT in the serial testing and its within-subject variability among HCWs is remarkably limited. The borderline zone of 4 to 8 SFCs propagated for the T-SPOT and following the US regulatory authority FDA (5 to 7 SFCs) is based mainly on a single published study and must be considered to be not yet sufficiently substantiated by scientific evidence, especially as the study by van Zyl-Smit et al. covered a follow-up observation period of only six months [20].

**Figure 2:**
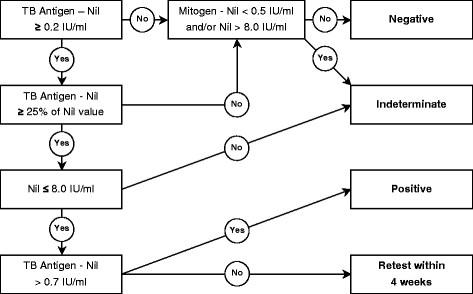
**Proposed flow chart for the interpretation of repeat QFT results in the serial testing of health care workers in countries with low or intermediate TB incidence rates.** Abbreviations: QFT = QuantiFERON-TB Gold In-Tube; TB = tuberculosis.

Hence, in view of the available data on the within-subject variability of the IFN-γ response and the use of IGRAs in the serial testing of HCWs in countries with low and intermediate incidences of TB, we recommend using a borderline zone from 0.2 to 0.7 IU/ml for the interpretation of repeat QFT results in routine screening of HCWs with an increased LTBI risk (Figure 2). Subjects with QFT results within this borderline zone, with suspected fresh infection due to their risk of TB exposure, and who are considered for preventive chemotherapy should be retested with the QFT within a period of about four weeks before preventive chemotherapy is recommended. If a HCW with a previously positive IGRA is to be retested in accordance with OSH regulations, repeating the IGRA appears appropriate. Only if repeat IGRA testing has demonstrated that the HCW remains persistently IGRA-positive with accordingly high IFN-γ responses should they be x-rayed immediately at their next TB screening in order to rule out active disease.

## Abbreviations

BCG = Bacillus Calmette-Guérin; HCW(s) = Health care worker(s); IFN-γ = Interferon-γ; IGRA(s) = Interferon-γ release assay(s); LTBI = Latent tuberculosis infection; OSH = Occupational safety and health; QFT = QuantiFERON-TB Gold In-Tube; SFCs = Spot-forming cells; TB = Tuberculosis; T-SPOT = T-SPOT.TB; TST = Tuberculin skin test; WHO = World Health Organization.

## Competing interests

The authors declare that they have no competing interests.

## Authors’ contributions

FCR, AS, and AN have made substantial contributions to the conception and design of the review, the acquisition and analysis of the review data, and have been involved in drafting and revising the manuscript. All authors have read and approved the final manuscript.
